# Tumour biomarkers—Tracing the molecular function and clinical implication

**DOI:** 10.1111/cpr.12589

**Published:** 2019-03-14

**Authors:** Jiahao Lin, Lie Ma, Di Zhang, Jiafeng Gao, Yipeng Jin, Zhihai Han, Degui Lin

**Affiliations:** ^1^ The Clinical Department, College of Veterinary Medicine China Agricultural University Beijing China; ^2^ Department of Respiratory Disease The Navy General Hospital of PLA Beijing China

**Keywords:** CTCs, ctDNAs, exosomes, miRNAs, proteins, tumour biomarkers

## Abstract

In recent years, with the increase in cancer mortality caused by metastasis, and with the development of individualized and precise medical treatment, early diagnosis with precision becomes the key to decrease the death rate. Since detecting tumour biomarkers in body fluids is the most non‐invasive way to identify the status of tumour development, it has been widely investigated for the usage in clinic. These biomarkers include different expression or mutation in microRNAs (miRNAs), circulating tumour DNAs (ctDNAs), proteins, exosomes and circulating tumour cells (CTCs). In the present article, we summarized and discussed some updated research on these biomarkers. We overviewed their biological functions and evaluated their multiple roles in human and small animal clinical treatment, including diagnosis of cancers, classification of cancers, prognostic and predictive values for therapy response, monitors for therapy efficacy, and anti‐cancer therapeutics. Biomarkers including different expression or mutation in miRNAs, ctDNAs, proteins, exosomes and CTCs provide more choice for early diagnosis of tumour detection at early stage before metastasis. Combination detection of these tumour biomarkers may provide higher accuracy at the lowest molecule combination number for tumour early detection. Moreover, tumour biomarkers can provide valuable suggestions for clinical anti‐cancer treatment and execute monitoring of treatment efficiency.

## 1 INTRODUCTION

Tumour biomarkers are molecules produced by tumour cells, which can indicate the biological status of tumour and can be used to evaluate the disease status and the efficiency of therapeutic interventions.

To survive and adapt in human and animal body, tumour cells have inherited genetic instability that leads to genetics alteration, including cancer‐specific mutations or changes in gene expression. These genetic alterations not only promote tumour development but provide researchers with a chance to chase the disease status at the same time. Although the term “tumour biomarker” now covers any molecular, biochemical, physiological, or anatomical property that reflects tumour's presence and status which can be quantified or measured, an ideal tumour biomarker is preferred to be collected non‐invasively from body fluids, such as the blood. These biomarkers include microRNAs, ctDNAs, proteins, exosomes and CTCs released by the tumour and circulating in the body fluids.

**Figure 1:**
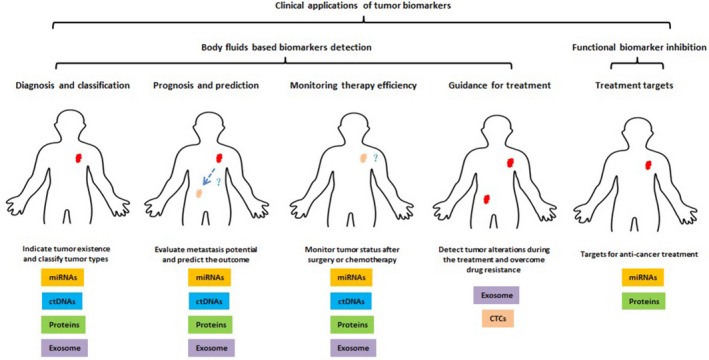
Clinical applications of tumour biomarker in different stage during cancer development and anti‐cancer treatment

Generally, tumour biomarkers are not expected to simply show the status of tumour, but to exhibit important functions for tumour's survival, growth and metastasis. Based on this fact, tumour biomarkers are recently regarded as treatment targets. Moreover, tumour biomarkers get an emerging role to direct the treatment of anti‐tumour drugs. In 2017, Food and Drug Administration (FDA) accelerated the approval of Keytruda (pembrolizumab), an antibody drug targeting PD‐1(programmed death 1), for the treatment of adult and paediatric patients with unresectable or metastatic solid tumours that have been identified as having a specific genetic feature (or tumour biomarker) referred to as microsatellite instability‐high (MSI‐H) or mismatch repair deficient (dMMR). Doctor Richard Pazdur, the acting director of the Office of Hematology and Oncology Products in the FDA's Center for Drug Evaluation and Research and director of the FDA's Oncology Center of Excellence, recommended this work as “this is an important first for the cancer community,” he said, “Until now, the FDA has approved cancer treatments based on where in the body the cancer started‐for example, lung or breast cancers. We have now approved a drug based on a tumor's biomarker without regard to the tumor's original location.” [https://www.fda.gov/newsevents/newsroom/pressannouncements/ucm560167.htm]. In this review, we will overview some current tumour biomarkers, discuss their biological functions, evaluate their roles in clinical treatment and compare the strength and limitations between different detected markers **(**Table 1 **)**, which may provide a prospect for the clinic applications of these markers during different stages of tumour development and anti‐cancer treatment **(**Figure 1 **)**.

**Table 1:** Comparison of different tumour biomarker detection methods for clinical applications

Biomarker	Modality	Strengths	Limitations	Ref.
Imaging‐based methods				
CT, MRI, PET, etc		High accuracy, displaying solid tumour visually	High ionizing radiation, unable to detect minimal tumours	183
Solid biopsy				
IHC staining, etc		Reflecting histological situations	Invasive detection methods, cannot cover all heterogeneity	124
Body fluids biopsy				
miRNAs	Altered level of tumour‐specific miRNAs, such as miR‐21 and miR‐155.	Non‐invasive, high sensitivity, allowing for early detection	Unstable, limited by individual difference	50‐52
ctDNAs	Tumour‐specific mutations, such as EGFR and BRAF.	Non‐invasive, high sensitivity, reflect individual difference, allowing for early detection	Lack of functional studies	199‐122
	DNA methylations, such as ALX4.			
Proteins	Elevated level of proteins, such as AFP and CA‐125.	Non‐invasive, high sensitivity, allowing for early detection	Limited by individual difference	116
	Different expression profiles, such as ER, PR, HER2, etc			
Exosomes	Increased exosome number	Non‐invasive, relatively stable in exosome, allowing for early detection	Limited isolation efficiency, lack of large scale studies	166‐168
	Different exosomal nucleotides and proteins			
CTCs	Increased CTC number	Non‐invasive, reflecting the evolutions of tumour cells timely during tumour development and treatment	Affected by isolation and selection methods, lack of large scale studies, can only be detectable during metastasis but can hardly be detected at an early stage	194‐196
	Altered nucleotides and proteins in CTCs			

The table shows the classification of currently used tumour biomarker detection methods and compared their strengths and limitations considering whether it is less harmful to patients, convenient to detect, with a high accuracy, high stability, can be detectable at an early stage, reflecting individual difference and indicating tumour evolution during development and treatment.

CT, computed tomography; IHC, immunohistochemistry; MRI, magnetic resonance imaging; PET, positron emission tomography.

## 2 THE MOLECULAR FUNCTIONS AND CLINICAL USE OF MIRNAS AS TUMOUR BIOMARKERS

### 2.1 The discovery of miRNAs as biomarker

miRNAs are small non‐coding RNAs (ncRNAs) that target corresponding messenger RNAs (mRNAs) to post‐transcriptionally downregulate certain gene expression. miRNAs were first identified in Caenorhabditis elegans in 1993,^1^ and extracellular miRNAs were first discovered in plants in 1996.^2^ Until now, over 2500 human miRNAs have been identified.^3^

### 2.2 Cancer‐related molecular functions of circulating miRNAs

The first study linking miRNA with cancer was published in 2002.^4^ After that, many groups focused their research on miRNA regulating cancer process and found that it involves in all hallmarks of cancer as defined by Hanahan and Weinberg.^5^ The functions of miRNAs can either be tumour supportive or tumour suppressive, often depending on the genes they targeted. For example, some best‐characterized cancer‐related microRNAs were listed below.

#### 2.2.1 Let‐7 family

The Let‐7 family include 13 different members and have been reported to be related with many types of cancer, and it was recognized as a tumour suppressor generally. Let‐7 regulates cancer cell cycle and proliferation by targetingRAS genes,^6^, ^7^ HMGA2,^8^, ^9^ STAT3,^10^ UHRF2^11^ and MYC,^12^, ^13^, ^14^ and additionally, it can regulate cell apoptosis by targeting CASP3.^15^

#### 2.2.2 miR‐15/16

miR‐15/16 is also an important tumour‐suppressing miRNA during various types of tumour progression. It can regulate apoptosis through targeting FEAT/METTL13,^16^ RPS6KB1, IGF1R,^17^ CCND1,^18^ BCL2,^19^ RECK and/or SOX6.^20^ It is a regulator of cell cycle process by targeting FG2F, CCNE1 and E2F1,^21^, ^22^, ^23^ and it is also involved in cell autophagy and metastasis by targeting mTORC2 and SOX5.^24^, ^25^

#### 2.2.3 miR‐21

The function of miR‐21 is mainly tumour promoting, since it targets many genes that are important tumour suppressors. These targeted genes mainly related to cell apoptosis, growth, invasion and tumour migration, such as BCL2,^26^ PTEN,^27^, ^28^ TP53, TGFB1,^29^ RECK,^30^ RHOB,^31^ TPM1^32^and PDCD4.^33^, ^34^

#### 2.2.4 The miR‐29 Family

Members of miR‐29 family usually act as tumour suppressors, and their downregulation always related to many types of cancer. They directly target cell cycle gene CDK6,^35^, ^36^, ^37^ apoptosis genes MCL1, BCL2 and FHIT,^35^, ^38^, ^39^ and migration and invasion genes LAMC1 and CDC42.^40^, ^41^

#### 2.2.5 The miR‐34 Family

ThemiR‐34 family are well known to regulate cell cycle, senescence, apoptosis and invasiveness in cancer. They target at genes that encode factors required for G1/S transition such as MYC, E2F, CDK4 and CDK6. They also target anti‐apoptotic genes such as BCL2, SIRT1 and genes involved in tumour cell invasion such as MET.^42^

#### 2.2.6 miR‐155

The genes targeted by miR‐155 are involved in multiple pathways related to multiple cancer‐related processes. For example, SMAD5 regulates the epithelial‐mesenchymal transition (EMT) process, while SOCS1, INPP5D and CSF1R regulate cell proliferation, and CASP3, FADD, APAF1 and FOXO3A regulate cell apoptosis.^43^, ^44^, ^45^, ^46^, ^47^, ^48^, ^49^

Currently, there are about thousands of studies about miRNAs as tumour biomarkers, including numerous reviews that have summarized the detail information about the history, classification and functions of tumour‐related miRNAs. Since tumour is of highly heterogeneity, different cancer types have different regulating molecule mechanisms, so some reviews also summarized the miRNA clinical usage by cancer types.^50^, ^51^, ^52^

### 2.3 Present applications of circulating miRNAs in clinic

In cancer patients, cancer‐related miRNAs will get some changes in expression or mutations and resulted in abnormal functions that facilitate cancer progression. The high‐throughput sequencing was applied to analyse the expression and mutation of miRNA genes and identified a series of aberrant expression profiles in many human cancers types, such as lymphoma,^53^ breast cancer,^54^ colorectal cancer,^55^ prostate cancer^56^ and glioma.^57^ These miRNAs change can be reflected in blood or other body fluid and FFPE tissues, and is even detectable in exosome or CTCs.

#### 2.3.1 Circulating miRNAs for the diagnosis of cancers

In plasma, the combination of miR‐21, miR‐145 and miR‐155 could help distinguish lung cancer patients with 69.4% sensitivity and 78.3% specificity.^58^ Combination of miR‐148b, miR‐409‐3p and miR‐801 could significantly distinguish breast cancer cases and healthy controls.^59^ In other body fluids, such as sputum, the combination of miR‐205, miR‐210 and miR‐708 distinguished lung squamous cell carcinoma patients with 73% sensitivity and 96% specificity.^60^

#### 2.3.2 Circulating miRNAs for the classification of cancers

The reason why miRNAs can be used for the classification of cancers is that different tissues have different miRNAs expression pattern, and miRNAs can reflect the origin of a specific type of tumour or even cellular subsets. In a blind study including 22 different tumour types, classifying tumours according to tissue of origin, the miRNA expression signatures can reach accuracy higher than 90%.^61^ Recent studies showed that distinct miRNA expression signatures could indicate different cellular subsets in acute myeloid leukaemia (AML)^62^ and prostate cancer.^63^ Since we are entering the era of personalized medicine, the anti‐cancer treatment for each patient increasingly depends on molecular analyses, which means establishing a classification according to miRNAs molecular functions that can direct clinic therapy is urgently in need.

#### 2.3.3 The prognostic and predictive values of circulating miRNA for therapy response

Since miRNAs have important regulatory functions during cancer development, their levels can reflect tumour status to some extent and thus could predict the outcome of therapy response. For example, low level of let‐7, a tumour suppressor miRNA, is correlated with poor prognosis including tumour size, overall survival and early recurrence. Moreover, the expression of miR‐21, a tumour‐promoting miRNA, is negatively correlated with relapse‐free survival of diffuse large B‐cell lymphoma (DLBCL) patients.^64^ In a study of 391 patients with advanced NSCLC, Wang et. al. found that high expression of miR‐16 was obviously associated with better survival.^65^

#### 2.3.4 Circulating miRNAs as monitors for therapy efficacy

In chronic myeloid leukaemia (CML), the level of cells with the BCR‐ABL rearrangement is widely used to characterize the disease progression, and decreases after imatinib treatment. It was reported that miR‐451 levels negatively correlate with BCR‐ABL levels and can monitor the therapy effect of imatinib at both the time of diagnosis and after treatment.^66^

#### 2.3.5 Circulating miRNA as targets for anti‐cancer therapeutics

The first microRNA‐based anti‐cancer therapy is MRX34, a synthetic miR‐34a mimic that is loaded into liposomal nanoparticles,^67^ which acts as a tumour suppressor miRNA downstream of p53. Another example is Miravirsen, a modified sequence complementary to miR‐122. Miravirsen was used for hepatitis C therapy and showed reduction in viral RNA with no evidence of resistance.^68^ Despite the different therapy effects of different miRNAs targets, the remaining problem is drug resistance, so developing a proper drug combination is one way to have better therapy outcomes.

## 3 THE MOLECULAR FUNCTIONS AND CLINICAL USE OF CTDNAS AS TUMOUR BIOMARKERS

### 3.1 The discovery of ctDNAs as biomarker

Cell‐free DNA (cfDNA) is small pieces of DNAs released into blood by various mechanisms mainly including cell apoptosis and necrosis. It was first identified in 1948 by Mandel and Metais in the blood of healthy people.^69^ Under normal conditions, cell‐free DNA levels are relatively low since apoptotic and necrotic cells are cleared by infiltrating phagocytes. For cancer patients, the cell‐free DNA fraction is often tumour cells derived, which is called circulating tumour DNA (ctDNA). ctDNAs are usually at a higher level with cancer patients and contain some genetic alterations specific for tumour cells.

### 3.2 Cancer‐associated genetic alterations of ctDNAs

ctDNAs were initially used to identify the presence of tumour in 1994, when Vasioukhin et al detected tumour‐specific RAS mutations in the plasma of cancer patients.^70^ Generally, ctDNA carries genomic and epigenomic information different from normal cfDNAs, such as point mutations, changed integrity, rearranged sequences, copy number variation (CNV), loss of heterozygosity (LOH), microsatellite instability (MSI) and DNA methylation.^71^

#### 3.2.1 Tumour‐specific genetic alterations

Abundant mutations have been detected in the ctDNAs of patients with various types of cancer. For example, PIK3CA mutations,^72^ HER2^73^ and ESR1^74^ higher amplification were detected in breast cancers patients. In colorectal cancers, tumour‐specific gene alterations of EGFR, BRAF, ALK, KIT, PDGFR, HER2 and KRAS^75^, ^76^, ^77^ were detected via ctDNA‐based assays. In the cases of lung cancers, EGFR mutations and ALK rearrangements were also identified.^78^, ^79^, ^80^ ctDNA concentration was significantly increased in other types of cancers such as periampullary cancer,^81^ oesophageal cancer,^82^ head and neck cancer,^83^ renal cancer,^84^ melanoma^85^ and prostate cancer.^86^ Besides, high LOH frequencies, particularly the observed CCND2 loss, were associated with the aggressiveness of breast cancer.^87^

#### 3.2.2 DNA methylation in ctDNA

DNA methylation plays important regulatory roles in gene expression and genome stability. For example, high levels of 5‐methylcytosine at the promoter region always result in gene transcriptionally silence. And methylation at the promoter region or non‐coding sequences is often dysregulated in many types of tumour and is associated with tumour initiation, progression, dissemination and metastasis.^88^ Some detectable ctDNA methylation in cancer patients consists of MLH1, CDKN2A (INK4A), ALX4, CDH4, NGFR, RUNX3, SEPT9, TMEFF2^89^, ^90^, ^91^, ^92^, ^93^, ^94^, ^95^and so on.

### 3.3 Present applications of ctDNAs in clinic

It is believed that cancers are results of gene mutation accumulation. These oncogenic genetic alterations can not only facilitate tumour progression and metastasis, but also be closely correlated with acquired treatment resistance.

#### 3.3.1 ctDNAs for the diagnosis of cancers

Present studies for ctDNAs used in diagnosis are lack of large scale study, and the specificity is not ideal enough for early diagnosis of certain types of cancers. This is partially because the detection methods at present are limited to detect the very low amount of ctDNAs in early stage of cancer patients, and some mutations such as KRAS are not specific for certain types of tumour but exist in many tumour types.^96^

Recently, Dennis Lo group use plasma Epstein‐Barr virus DNA to screen for nasopharyngeal cancer. In the study, a total of 20,174 participants underwent nasopharyngeal cancer screening, and the sensitivity and specificity were 97.1% and 98.6%, respectively.^97^ This study may provide us a new sight about detecting cell‐free DNAs, not simply limited by tumour secreted factors but also include those factors that cause the cancer.

#### 3.3.2 The prognostic and predictive values of ctDNAs

Some patients cured by surgery still receive adjuvant chemotherapy in case of tumour relapse. Studies showed that detecting ctDNAs before and after surgical resection can identify individuals with residual disease,^98^ and predict disease recurrence.^83^, ^84^ For example, the high concentration of ctDNA is positively correlated with a poorer survival in metastatic colorectal cancers with detectable KRAS ctDNA.^99^ Another example is that detecting the methylation of MGMT promoter region on ctDNAs in glioblastoma multiforme patients can also direct whether it is necessary to have adjuvant treatment after surgery.^100^, ^101^ Nowadays, the prognostic and predictive value of ctDNA has been extended to different type of cancers, such as cervical cancer,^102^ colorectal cancer,^103^, ^104^ pancreatic cancer,^105^, ^106^, ^107^ melanoma^108^, ^109^ and breast cancer,^110^, ^111^ in which the increased levels of ctDNA are related to poor overall survival.

#### 3.3.3 ctDNAs as monitors for therapy efficacy

The level of ctDNA is closely correlated with tumour burden and therapeutic responses. It has been reported that its levels increased rapidly with disease progression and declined correspondingly after successful treatment in melanoma,^109^, ^112^ breast,^110^ ovarian^113^ and colon cancers.^114^, ^115^

#### 3.3.4 ctDNAs as guidance for treatments

Recently, there are many new methods for the detection of ctDNA to monitor emerging resistant mutations during anti‐cancer treatment, which allow us to choose appropriate treatment based on specific mutations detected in the drug‐resistant tumour for each individual. For example, in colorectal cancer patients undergoing anti‐EGFR treatment, detecting KRAS mutations in ctDNAs of patients with anti‐EGFR therapies can identify relapse10 months before radiographic documentation of disease progression.^116^ Similar situations also include BRAF L597 mutation in cutaneous melanoma with MEK inhibitor and PIK3CA mutation in solid tumours with PIK3CA inhibitors.^117^, ^118^

There are many more reports which introduce ctDNAs as cancer biomarkers at different aspects; a selection of reviews^119^, ^120^, ^121^, ^122^may also serve as a starting point for readers outside the field.

## 4 THE MOLECULAR FUNCTIONS AND CLINICAL USE OF PROTEINS AS TUMOUR BIOMARKERS

Compared with other types of tumour biomarkers, cancer‐related proteins are earlier and more widely used in the clinic. Until now, numerous proteins have been identified to be upregulated with tumour burden, which can either be detectable in tumour tissues or in patients’ blood.

### 4.1 Cancer‐associated protein markers

#### 4.1.1 Present protein markers in clinic

At present, American National Cancer Institute lists the protein tumour markers that are now used in clinic, for example, alpha‐fetoprotein (AFP) for liver cancer and germ cell tumours, CA15‐3 for breast cancer, CA19‐9 for pancreatic cancer and gastric cancer, CA‐125 for ovarian cancer, carcinoembryonic antigen (CEA) for colorectal cancer and some other cancers, and so on. Other protein biomarkers also include calcitonin for medullary thyroid cancer, CD20 for non‐Hodgkin lymphoma, chromogranin A (CgA) for neuroendocrine tumours, beta‐2‐microglobulin (B2M) for multiple myeloma, and so on.

#### 4.1.2 Other potential markers to be used in clinic

In breast cancer, there is a clear molecular subtype based on some important protein such as ER, PR and HER2, whose function is correlated with breast cancer progression. And the combination of several genes was also commercially used to predict the clinical outcome of breast cancer patients.^123^ Another widely used marker is EGFR mutations for lung cancer, whose function is closely related to tumour progression through important signalling pathways such as MAPK and AKT/PI3K, and there are also many clinical drugs targeting EGFR.^124^

### 4.2 Present applications of cancer biomarker proteins in clinic

#### 4.2.1 Protein markers for the diagnosis of cancers

At present, the accuracy of a single protein biomarker can only discriminate cancer patients and healthy individuals, which is certainly not enough for early diagnosis and further clinical use. Currently, only few body fluid‐based protein markers were approved by FDA, and none of them have high accuracy for early clinical diagnosis. One promising way to increase the accuracy for disease diagnosis is to combine several protein markers. For example, the OVA1 test for ovarian cancer identified five protein markers in serum including CA125, transthyretin, apolipoprotein A‐I (APOA1), β2‐microglobulin and transferrin^125^, ^126^; the combination of these five proteins has a ROC AUC of 0.90 and predicts 91.4% ovarian malignancy in the cases of early‐stage disease. This result shows a dramatic accuracy improvement compared with 65.7% for CA125 alone.^127^, ^128^

#### 4.2.2 The prognostic and predictive values of protein markers

In breast cancer patients, 21 proteins were identified from an antibody microarray containing 135 antibody fragments, whose functions related to the development of metastasis. The combination of these 21 proteins could distinguish patients at high or low risk for developing metastasis, with an ROC AUC of 0.85.^129^ What is more, this 21 proteins combination also provided an added value to clinic. That is when combined with conventional clinical parameters, which the ROC AUC is 0.66, the ROC AUC could increase to 0.90 for prediction of recurrence.^129^

#### 4.2.3 Protein markers as monitors for therapy efficacy

Another function that is widely used in clinic is to monitor the therapy efficacy. Besides some traditional tumour biomarkers such as CA125, many studies work to identify other new tumour biomarkers in different cancer types to improve the present situation. For example, the level of a newly identified tumour biomarker Hsp90α in patients’ plasma showed significant correlation with therapy efficacy in lung cancer.^130^

## 5 THE MOLECULAR FUNCTIONS AND CLINICAL USE OF EXOSOMES AS TUMOUR BIOMARKER

### 5.1 The discovery of tumour‐derived exosomes as tumour biomarkers

Exosomes are 30‐100 nm small vesicles secreted by cells to the extracellular matrix or body fluids. It was first discovered in 1980s by Johnstone group who declared that transferrin receptor could be selectively released in circulating vesicles, which was later named exosomes.^131^, ^132^, ^133^ In 1990s, people recognized exosomes to be related with immune system functions.^134^ In 2010s, researchers found that exosomes contain RNAs, DNAs, proteins and metabolites.^135^ And in recent years, exosomes were found to have an important role in cell‐cell communication and signalling transduction.

### 5.2 Functions of exosomes during cancer development

Exosomes play an important role in cell‐cell communication, since exosomes can package certain RNAs, DNAs, proteins and other metabolism from the donor cells. There are emerging evidences that tumour cells secret exosomes to facilitate cancer growth, angiogenesis, invasion, metastasis, immunity and even drug resistance acquirement.

#### 5.2.1 Exosomal Nucleic Acids

The nucleic acids in exosomes include RNAs such as miRNAs, mRNAs, tRNAs, lncRNAs^136^, ^137^, ^138^, ^139^ and DNAs including single stranded and double stranded.^135^, ^140^ Among these nucleic acids, exosomal miRNAs draw most of the attention.^141^, ^142^, ^143^ It has been reported that pro‐angiogenic miRNAs within tumour‐derived exosomes can induce angiogenesis.^144^ Furthermore, miRNAs, such as miR21 and miR29a that are highly expressed in tumour cells, can be transported by exosomes and bind to toll‐like receptors to trigger the inflammatory response, which will facilitate tumour growth and metastasis.^145^ Cancer cells can also gain miRNAs information from other cancer‐associated cells. For example, miR21 could help to suppress ovarian cancer apoptosis and bind to apoptotic protease‐activating factor‐1 (APAF1) to confer drug resistance to paclitaxel.^146^

#### 5.2.2 Exosomal Proteins

Tumour‐derived exosomes can transport many proteins to establish a complex metastatic microenvironment. For example, HSP70, HSP90 and survivin can inhibit apoptosis and promote cellular proliferation.^147^, ^148^ VEGF, FGF and TGF‐β were reported to facilitate angiogenesis.^149^ Tumour‐derived exosomes were enriched with MMPs (such as MMP‐1 and MMP‐19),^150^ which could degrade ECM components to facilitate cancer invasion.^151^ Recently, exosomes were found to inhibit immune system to promote tumour development by increasing the immune suppressive cells, decreasing NK and T cells proliferation and cytotoxicity, inhibiting antigen‐presenting cell number and function.^152^, ^153^, ^154^ There are emerging reports that tumour‐derived exosome also mediates the acquirement of drug resistance. One example is that exosomes of HER2‐overexpressed breast cancer cells also contain HER2 molecules, which can be combined with the HER2 antibody drug trastuzumab, thus prevent the drug from binding to tumour cells and inhibit the anti‐tumour effects.^155^

### 5.3 Present applications of tumour‐derived exosomes in clinic

#### 5.3.1 Tumour‐derived exosomes for the diagnosis of cancers

One advantage for exosomal markers in clinical detection is its stability to avoid from enzyme‐based degradation compared with circulating markers, which may increase the accuracy of the detection. For example, miRNA‐1246 shows a sensitivity of 71.3% and a specificity of 73.9% for the diagnosis of oesophageal squamous cell cancer (ESCC), and its level is also correlated with the tumour metastasis and poor survival.^156^ Actually, many circulating cell‐free biomarkers can also be detected in exosomes in many types of cancer. For example, oncogene EGFR also exists in exosomes from prostate cancer patients.^157^ miRNAs such as miR‐21 and miR‐141, which was previously known as diagnostic markers for ovarian cancer, were also present in exosomes from ovarian cancer patients.^141^ KRAS and p53 mutations in exosomal DNA of pancreatic cancer also could predict the treatment option and therapy resistance.^158^

#### 5.3.2 The prognostic and predictive values of tumour‐derived exosomes

Similar to the diagnosis property, exosomal biomarker also shows values in prognosis and prediction. For example, in tongue squamous cell carcinoma (TSCC), the higher level of caveolin‐1 (CAV1) in exosomes is negatively correlated with recurrence and survival.^159^ In nasopharyngeal carcinoma (NPC), the level of miR‐24‐3p was higher in exosomes from patients compared with healthy people and was correlated with lower disease‐free survival.^154^

#### 5.3.3 Tumour‐derived exosomes as monitors for therapy efficacy

It was reported that the amount of cisplatin in exosomes released from cisplatin‐resistant cells is 2.6 times higher than that from cisplatin‐sensitive cells after treatment with cisplatin.^160^

#### 5.3.4 Tumour‐derived exosomes in anti‐cancer therapy

Since exosomes play an important role in cancer growth, metastasis and drug resistance, some drugs target to inhibit the secretion of exosomes or just remove exosomes from blood circulation.^161^, ^162^ Since exosomes can protect its contents from degradation by enzymes, they are ideal drug delivery vehicles, especially for delivering some suppressor miRNAs such as miR‐143.^163^, ^164^ Because of the role of exosomes in the immune system, exosome could represent new antigens to the immune cells to evoke the immune system and finally overcome the immune escape of tumour cells.^165^

Although many challenges exist, it is still a promising field of biomarker, and interested readers are referred to some excellent reviews.^166^, ^167^, ^168^

## 6 THE MOLECULAR FUNCTIONS AND CLINICAL USE OF CTCS AS TUMOUR BIOMARKERS

### 6.1 The discovery of CTCs as tumour biomarkers

CTCs are tumour cells from a primary tumour that circulate in the blood around the body, and act as seeds for subsequent secondary metastatic tumour at distant organs. The amount of CTCs in blood is very low at about 1‐10 CTCs per mL of whole blood in metastatic patients.^169^ CTCs were first identified in 1869.^170^ Recent years, with the development of CTCs isolation and detection techniques, CTCs have been investigated as promising clinical tumour biomarkers in numerous types of cancer.

### 6.2 Functions of CTCs during cancer development

Since studies have recognized that CTCs are heterogeneous, which means a CTC cluster may not only contain different sizes or components of CTCs, but also include tumour‐associated stromal cells. These certain different characteristics have distinct biological functions and higher metastatic potential,^171^, ^172^, ^173^ and CTC clusters have higher metastatic potential (near 100‐fold) compared to individual CTCs.^174^ For example, the formation of CTC clusters requires protein expression such as plakoglobin and keratin 14, which are related to tumour metastases.^171^, ^175^ Some factors in the circulation microenvironment also participate in CTC metastasis ability such as pro‐inflammatory cytokines system.^176^ The presence of stromal cells such as endothelial cell and platelets also facilitates CTC cluster metastasis through different mechanisms.^177^, ^178^ What is more, bigger size CTC clusters are under more hypoxia conditions and are more potent to metastasis.^179^

### 6.3 Present applications of CTCs in clinic

#### 6.3.1 CTCs for the diagnosis of cancers

The origin and function of CTCs determine that it can be detected in patients already undergoing metastasis. But for early‐stage non‐metastasis patients, it can rarely be detected in the circulation, which may limit its sensitivity and specificity for cancer diagnosis. However, in some cases, it can be used to distinguish lung cancer from benign lesions in patients at CTC count over 25.^180^ It can also be used for cancer screening, in tobacco‐induced chronic obstructive pulmonary disease, which are at high risk of developing lung cancer, only patients with detectable CTCs were diagnosed lung cancer later.^181^

#### 6.3.2 The prognostic and predictive values of CTCs

The numbers and characteristics of CTCs are getting widely studied for the use of survival prognosis or therapy response prediction. For example, in metastasis breast cancer, 46.9% of the patients had higher CTC level (≥ 5 CTCs/7.5 mL), which meant lower progression‐free survival and overall survival compared to patients with lower CTC number (<5 CTCs/7.5 mL).^182^ What is more, CTCs that are undergoing EMT with the expression of EMT marker plastin‐3 could also predict therapy outcome.^183^

#### 6.3.3 CTCs as monitors for therapy efficacy

Patients with lower CTC level after certain treatment exhibit better survival compared to those patients remain high CTC level. For example, in metastatic breast cancer, after one cycle of chemotherapy, patients with decreasing CTC levels have a better prognosis than patients with persistently high CTC levels.^184^ Similar results were also reported in colon cancer,^185^ castration‐resistant prostate cancer,^186^, ^187^ rectal cancer^188^, ^189^ and small cell lung cancer.^190^ In ovarian cancer, monitoring CTC has an even higher accuracy than protein marker CA125 for predicting chemotherapy response and cancer relapse.^191^

#### 6.3.4 CTCs as guidance for treatments

The response of tumour cells to therapy can be dynamic; thus, measuring CTCs during the course of therapy may reveal tumour changes timely and provide us guides for further treatment. However, until now, only limited studies showed CTC‐based treatment direction applicable. For example, breast cancer patients with HER2‐negative setting but HER2‐positive CTCs were treated with trastuzumab or observation. The results showed that 27 of 36 patients treated with trastuzumab became CK19 mRNA‐negative compared to 7 of 39 observation patients, and trastuzumab treatment also decreased the risk of disease recurrence and prolonged disease‐free survival.^192^ In a recent clinical trial investigating the clinical utility of CTC numbers in ER‐positive metastatic breast cancer patients, patients with low CTC numbers are given hormone therapy, while patients with high CTC numbers are treated by first‐line chemotherapy. After one cycle of chemotherapy, patients remaining high CTC numbers (>5 CTCs/7.5 mL) were possibly switched to second‐line chemotherapy at earlier time. However, until now, this study was reported as negative, since an early switch of chemotherapy did not improve overall survival of these patients.^193^ Many reasons may cause this result; however, based on the property of CTCs, it is still believed to be the future direction for guiding treatment timely, and new effective treatment is also urgently needed after identifying the CTC status.

Some reviews that summarized the advantages and challenges in this field also listed here for readers who are interested.^194^, ^195^, ^196^

## 7 CONCLUSIONS

Large portions of cancer death are caused by metastasis, so early diagnosis becomes the key to decrease the death rate. Since detecting tumour biomarkers in body fluids is the most non‐invasive way to identify the status of tumour development, it has been widely investigated for the use in clinic. These biomarkers include different expression or mutation in miRNAs, ctDNAs, proteins, exosomes and CTCs. For the use of early diagnosis, which requires the detection of tumour markers at early stage before metastasis, high sensitivity and specificity are very important. Due to the characteristic of CTCs, which are often detected when metastasis already happened, the sensitivity and specificity for early diagnosis are not high enough to be ideal prospective marker for diagnosis. Other molecular markers such as miRNAs, ctDNAs and proteins can be secreted by tumour cells at very early stage to facilitate tumour development and metastasis, thus could be detected at an early stage (Figure 1). However, at present, the sensitivity and specificity of these molecular markers are not high enough, which may partially due to the present limited detection methods, limited stability or may be limited by the different molecular function of different patients. So, one promising way to solve this problem is to combine these biomarkers and achieve a highest accuracy at the lowest molecule combination number for tumour early detection. Recently, exosomes are identified as an emerging hot spot in the field of diagnostic tumour biomarker, because circulating exosomes shows important functions in long distance message transport between different cells, and detecting miRNAs, ctDNAs and proteins in exosomes can avoid enzyme‐based degradation of these molecules, which allow an elevated accuracy (Figure 1). However, methods for detection of isolate exosomes and molecular marker combinations still need further study, especially considering their biological functions.

After the diagnosis of cancer, patients usually need certain anti‐cancer treatment. Another important function for tumour biomarkers is to direct the determination of therapeutic regimen. Many oncogenic miRNAs, ctDNAs and proteins have functions to facilitate cancer progression and metastasis, so they usually correlate with poor prognosis (Figure 1). CTCs, which often occurred just before metastasis, possess ideal accuracy for prognosis and treatment efficiency prediction. These may provide some information for choosing certain anti‐cancer treatment. Further studies should also focus on biomarkers‐targeted treatments based on their molecular functions, which could have a more precise direction for drug treatment.

Another important clinic use for tumour biomarker is to monitor the treatment efficiency. Since the tumour markers could reflect the status of tumour development, these levels could evaluate whether the treatment is effective to inhibit tumour development. Meanwhile, it can monitor whether there is a relapse after a period of remission (Figure 1). However, the accuracy for this use is limited and required more efficient alternative treatments if the first‐line treatment is not effective.

During the anti‐cancer treatment, tumour cells may evolve new properties to gain drug resistance. These properties can be reflected in the genomic change of metastasis tumour cells, and the genetic information in CTCs provides an ideal way to identify these changes (Figure 1). At present, the study of this application is only at a very beginning stage, and the clinical results for CTCs directed treatment changes cannot improve their overall survival of these drug resistance patients. It may because that the alternative treatment is not effective. And the method for isolating CTCs is not mature, since different CTCs have different surface markers, and present method may not isolate all kinds of CTCs in the circulation.

At present, traditional tumour biomarkers widely used in clinic are still at protein level. However, due to their limited sensitivity and specificity, novel serum biomarkers such as CTCs and nucleic acids will have a great advantage in the future. Although, novel biomarkers have their own technical limitation, and protein markers may not soon be replaced, it is a trend that using different biomarkers in combination to increase the sensitivity and specificity. In the era of individualized medical treatment and precise medical treatment, the diagnosis and treatment decision relied much on the information provided by tumour biomarkers. One important event for this era is that in this year, FDA first approved a drug based on a tumour's biomarker without regard to the tumour's original location. And we believe that researchers will pay more attention on the molecular functions and the underlying mechanisms of these tumour biomarkers, to have a more precise use in the clinics in the future.

## ETHICS APPROVAL AND CONSENT TO PARTICIPATE

Not applicable.

## CONSENT FOR PUBLICATION

Not applicable.

## CONFLICT OF INTEREST

The authors declare that they have no competing interests.

## AUTHORS’ CONTRIBUTIONS

JHL and LM wrote the manuscript and created the figures. DZ and JFG collected the related paper. YPJ, ZHH and DGL provided guidance and revised this manuscript. All authors approved the final manuscript.
